# Factors Associated with the Timing of Liver Metastasis After Colorectal Cancer Surgery: A Retrospective Multicenter Study

**DOI:** 10.3390/cancers18152445

**Published:** 2026-07-29

**Authors:** Xinliang Liu, Cheng Zhou, Wenlong Qiu, Zongqi Li, Fangze Wei, Tixian Xiao, Shiwen Mei, Fei Huang, Fuqiang Zhao, Qian Liu

**Affiliations:** 1 Department of Surgical Oncology and General Surgery, The First Affiliated Hospital of China Medical University, Shenyang 110001, China; xinlianglsd@163.com (X.L.); 18840050467@163.com (Z.L.); 2 Department of Colorectal Surgery, National Cancer Center/National Clinical Research Center for Cancer/Cancer Hospital, Chinese Academy of Medical Sciences and Peking Union Medical College, Beijing 100021, China; b2024003031@student.pumc.edu.cn (C.Z.); 15154117801@163.com (W.Q.); wfzpumch@163.com (F.W.); b2023003127@pumc.edu.cn (T.X.); mswbxw@163.com (S.M.); frankhuangxm@outlook.com (F.H.); zhaofqncc@163.com (F.Z.)

**Keywords:** colorectal cancer, colorectal liver metastasis, early metachronous liver metastasis, late metachronous liver metastasis, KRAS mutation, BRAF^V600E^ mutation, associated factors

## Abstract

**Purpose**: This study aimed to determine the optimal temporal threshold for distinguishing “early” from “late” liver metastasis in patients who developed liver metastasis after colorectal cancer (CRC) surgery, and to evaluate whether KRAS and BRAF^V600E^ mutations, along with other clinicopathological factors, are associated with the timing of liver metastasis. **Methods**: This retrospective study utilized clinical and pathological data from patients who developed liver metastasis after radical CRC surgery at two centers from 2019 to 2023. X-tile software was used to identify the optimal temporal threshold. Logistic regression analysis was applied to determine if KRAS/BRAF^V600E^ mutations and other potential factors are independently associated with the time to onset of liver metastasis. **Results**: X-tile analysis identified 11 months post-surgery as the optimal cutoff for distinguishing early metachronous liver metastasis (EMLM) from late metachronous liver metastasis (LMLM), classifying 114 cases into the EMLM group and 72 into the LMLM group. Comparative analysis indicated statistically significant differences between the two groups in lymphovascular tumor emboli, perineural invasion, and postoperative adjuvant therapy (*p* < 0.05). Logistic regression analysis revealed that neither KRAS mutation (OR, 1.185; 95% CI: 0.641–2.190; *p* = 0.587) nor BRAF^V600E^ mutation (OR, 2.836; 95% CI: 0.302–26.642; *p* = 0.363) was independently associated with the timing of liver metastasis. In contrast, postoperative adjuvant therapy showed a statistical association with a likelihood of LMLM (OR, 0.253; 95% CI: 0.105–0.611; *p* = 0.002). **Conclusions**: This study identified 11 months post-CRC surgery as the optimal cutoff for differentiating EMLM versus LMLM. In this cohort, no statistically significant association was observed between KRAS/BRAF^V600E^ mutations and the timing of liver metastasis, whereas postoperative adjuvant therapy was statistically correlated with the likelihood of LMLM. This stratification may guide personalized surveillance strategies and provide valuable insights for future mechanistic investigations into the temporal heterogeneity of post-surgical liver metastasis. However, the interpretation and generalization of the findings require external validation in prospective cohorts.

## Simple Summary

Approximately 25% of patients with colorectal cancer (CRC) develop metachronous liver metastasis (MLM) after curative surgery, with earlier onset portending a worse prognosis. Current definitions distinguishing “early” from “late” MLM, however, are largely based on empirical convention or fixed follow-up intervals rather than on objective prognosis-derived inference. To address this gap, this study aimed to define a survival-based threshold for differentiating early versus late MLM, and to investigate whether KRAS/BRAF^V600E^ mutations and other clinicopathological factors are associated with the time to onset of liver metastasis after CRC surgery. In a cohort of 186 CRC patients who develop liver metastasis after radical surgery, we identified 11 months post-surgery as the optimal temporal cutoff, and found that KRAS/BRAF^V600E^ mutations were not significantly associated with the timing of liver metastasis. In contrast, postoperative adjuvant therapy was significantly correlated with it. Collectively, this temporal cutoff identified in this study facilitates postoperative risk stratification and, when combined with routine clinicopathological features, may serve as a pragmatic benchmark for guiding intensified surveillance protocols. Furthermore, the associated factors discussed in this study offer valuable insights for future mechanistic investigations into the temporal heterogeneity of post-surgical liver metastasis.

## 1. Introduction

Cancer remains a formidable challenge to global public health systems [1], with colorectal cancer (CRC) constituting a particularly critical subset due to its high incidence and mortality rates [2]. Liver metastasis is the leading cause of death among CRC patients [3]. Clinically, approximately 45% of CRC patients eventually develop liver metastasis, among whom about 20% present with synchronous liver metastasis (SLM) at the time of initial diagnosis, and an additional 25% develop metachronous liver metastasis (MLM) following curative surgery [4].

Importantly, emerging evidence suggests that survival outcomes for MLM may not be inferior to those for SLM [5,6]. Meanwhile, early metachronous liver metastasis (EMLM) is consistently associated with worse outcomes, characterized by rapid disease progression and resistance to standard therapies [7]. Nevertheless, no unified consensus has yet been reached within the academic and clinical communities regarding the optimal time point for defining “early” versus “late” liver metastasis [8]. Current classifications largely rely on arbitrary cutoffs based on empirical convention or fixed follow-up intervals, rather than on objective statistical inference derived from patient survival data [9].

Furthermore, the pathophysiological mechanisms governing the timing of metastatic seeding remain poorly delineated [10]. Conventional wisdom posits that specific driver mutations (e.g., KRAS, BRAF, TP53, etc.) confer an enhanced intrinsic ability for hematogenous dissemination [11,12]. RAS is one of the most frequently mutated oncogenes in CRC, with approximately 40% of patients harboring activating missense mutations in KRAS [13]. BRAF mutations, predominantly the V600E variant, are present in up to 10% of CRC patients [14]. Prior studies have revealed that RAS and BRAF mutations are associated with poor clinical outcomes in patients with colorectal liver metastasis (CRLM), with the BRAF^V600E^ mutation in particular serving as a poorer prognostic factor for overall survival (OS) [15,16]. Accordingly, a critical unresolved question is whether RAS and BRAF mutations are associated with the timing of liver metastasis and thereby contribute to the observed prognostic disparity.

To address this knowledge gap, this study aimed to: (1) determine the optimal cutoff for differentiating early from late metachronous liver metastasis (LMLM) based on objective survival data, and (2) evaluate the association of KRAS/BRAF^V600E^ mutations and other potential clinicopathological factors with the timing of liver metastasis in patients who developed liver metastasis after CRC surgery. These findings are expected to provide novel clinical insights for risk-adapted surveillance and future mechanistic investigations into the temporal heterogeneity of post-surgical liver metastasis.

## 2. Materials and Methods

### 2.1. Patients

This multicenter retrospective study was conducted at two institutions: the National Cancer Center/Cancer Hospital, Chinese Academy of Medical Sciences (Beijing, China), and the First Affiliated Hospital of China Medical University (Shenyang, China). The study cohort consisted of CRC patients who developed liver metastasis after curative surgery between 2019 and 2023, and for whom complete KRAS/BRAF^V600E^ mutation testing results were available. All enrolled patients met the following inclusion criteria:

(1) Pathologically confirmed diagnosis of CRC;
(2) Underwent radical CRC surgery at either participating institution;
(3) Postoperative liver metastasis confirmed by pathological examination or imaging studies.

The exclusion criteria were:

(1) Primary CRC was pathologically confirmed as non-adenocarcinoma, such as neuroendocrine carcinoma;
(2) Presence of distant metastasis prior to surgery;
(3) Non-radical surgery;
(4) Indeterminate liver metastasis status;
(5) Development of extrahepatic distant metastasis after CRC surgery;
(6) Incomplete clinicopathological data.

### 2.2. Sample Collection and Molecular Profiling

This study utilized CRC tumor samples obtained from surgical specimens. All samples were fixed with formalin and embedded in paraffin by experienced pathologists at two medical centers. All CRC tumors were tested for deficient mismatch repair (dMMR) via immunohistochemistry or microsatellite instability-high (MSI-H) via polymerase chain reaction (PCR). RAS/BRAF mutation profiling was performed using PCR or next-generation sequencing (NGS) assays. Given the considerable time span and the medical disparities between the two centers, the detection platforms and the panel of mutations assessed were not uniform. Therefore, the analysis was restricted to the clinically prevalent KRAS mutation and the BRAF^V600E^ variant. The molecular-testing details are provided in Table S1.

### 2.3. Definitions

Anatomically, radical surgery for colon cancer is defined as complete colectomy with systematic regional lymphadenectomy [17]. For rectal cancer, it refers to either total mesorectal excision or extended mesocolic excision [18]. MLM was defined as liver metastasis detected after CRC surgery [5]. All cases were staged per the 8th edition of the American Joint Committee on Cancer (AJCC) Cancer Staging Manual TNM classification system. OS was defined as time interval from surgical resection to date of mortality or last clinical follow-up. Metastasis-free survival (MFS) was defined as time interval from surgical resection to the time of liver metastasis, death, or the last follow-up. The last follow-up date was 1 January 2025.

### 2.4. Statistical Analyses

X-tile software (Robert L. Camp, M.D., Ph.D., Yale University, version 3.6.1) was used to identify the optimal cutoff for differentiating EMLM from LMLM based on OS differences. Comprehensive statistical analyses were performed using SPSS 26.0 software (Armonk, NY, USA) and GraphPad Prism 8 (La Jolla, CA, USA).

Continuous variables with normal distribution were described as mean ± standard deviation (SD) and compared using the Student *t*-test. Continuous variables with a skewed distribution were described as median with interquartile range (IQR) and compared using the Mann–Whitney U test. Categorical variables with unordered categories were described as frequency with percentage and compared using the chi-square test or Fisher’s exact probability test. All statistical tests were two-sided, and a *p*-value lower than 0.05 was considered statistically significant. To prevent the omission of potentially significant risk factors, the significance level for univariable analysis was set at 0.1 [19], and for multivariable analysis it was set at 0.05. Variables with *p* < 0.1 in the univariable analysis were entered into the multivariable logistic regression model. However, based on a priori clinical and biological knowledge, the primary exposure variables—KRAS and BRAF^V600E^ mutations—were forced into the multivariable model irrespective of their univariable significance. Collinearity among variables was assessed using variance inflation factors (VIFs), with VIF > 10 indicating significant collinearity.

## 3. Results

### 3.1. Patient Characteristics

**Figure 1:**
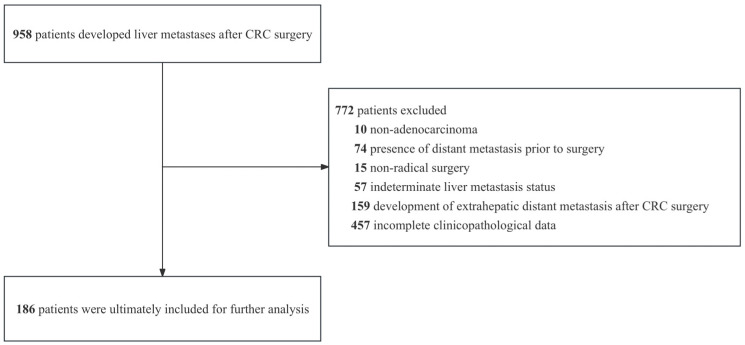
Flowchart of included patients. **Abbreviations**: CRC, colorectal cancer.

A total of 186 MLM patients with complete KRAS/BRAF^V600E^ mutational profiling were ultimately enrolled for further analysis (Figure 1). The clinicopathological baseline characteristics of the 186 included patients are shown in Table 1. The median age was 63 years (SD, ±10); 10 patients (5.4%) had a family history of CRC; 87 patients (46.8%) had concomitant diseases (liver cirrhosis, diabetes, cardiovascular and cerebrovascular diseases, hypertension, and chronic obstructive pulmonary disease); primary tumors were located in the right colon in 35 patients (18.82%), left colon in 49 patients (26.34%), and rectum in 102 patients (54.84%); 95 patients (51.1%) received neoadjuvant therapy prior to surgery; 112 patients (60.2%) had lymphovascular tumor emboli; 120 patients (64.5%) exhibited perineural invasion; regional lymph node metastasis was present in 139 patients (74.7%); dMMR/MSI-H was observed in 5 patients (2.7%); KRAS mutation was detected in 81 patients (43.5%); the BRAF^V600E^ mutation was identified in 6 patients (3.2%); concurrent wild-type status of RAS and BRAF was present in 99 patients (53.2%); postoperative adjuvant therapy was administered to 140 patients (75.3%); preoperative carcinoembryonic antigen (CEA) levels had a median value of 4.57 (IQR, 2.52–16.41) ng/mL; and preoperative carbohydrate antigen 19-9 (CA19-9) levels had a median value of 15.94 (IQR, 8.44–27.31) U/mL. Median follow-up was 37 (IQR, 21–52) months. During follow-up, 66 deaths (35.5%) were recorded.

### 3.2. The Optimal Cutoff for Differentiating EMLM from LMLM

**Figure 2:**
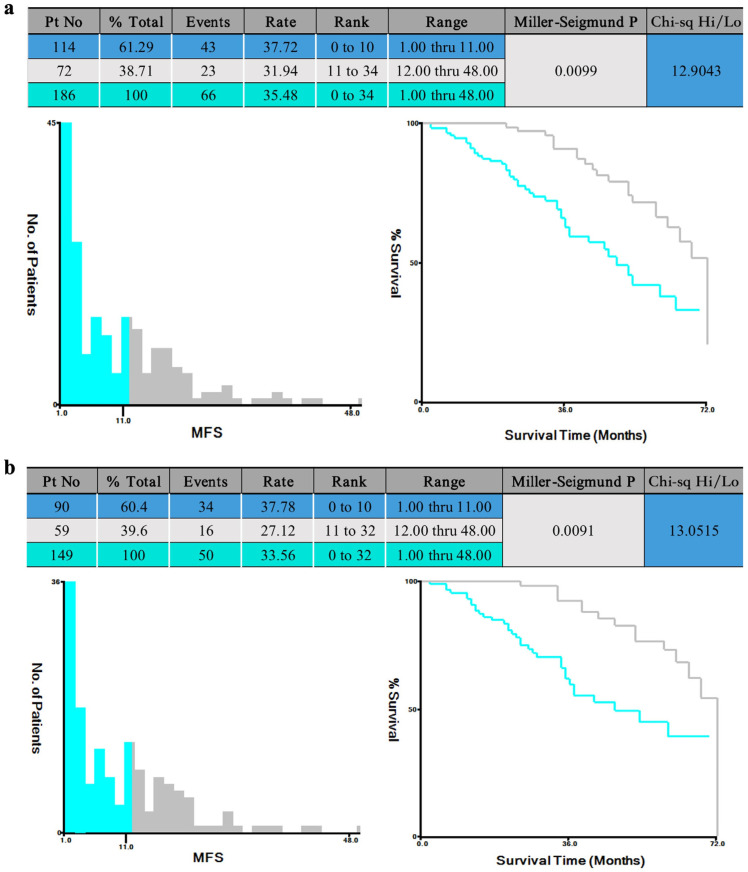
X-tile analysis determined 11 months as the optimal cutoff. (**a**) In the derivation cohort of 186 patients, X-tile analysis calculated an optimal MFS of 11 months. Based on this cutoff, patients were divided into EMLM and LMLM groups. The patient distribution and the survival curves for the two groups are presented (blue legend for EMLM; gray legend for LMLM). (**b**) In the validation cohort of 149 patients, the software again yielded an optimal cutoff of 11 months. After grouping, the patient distribution and the survival curves are also shown (blue legend for EMLM; gray legend for MLM). **Abbreviations**: MFS, metastasis-free survival; EMLM, early metachronous liver metastasis; LMLM, late metachronous liver metastasis.

When OS was used as the dependent variable and MFS as the independent variable in X-tile software, the optimal cutoff calculated by the software was 11 months (Figure 2a). This indicates that when defining 11 months post-surgery as the cutoff, the OS difference between EMLM and LMLM is maximized, with a *p*-value of less than 0.01. As no external independent validation cohort was available, 80% (149 of 186) of the total patient population were randomly allocated without replacement to form the internal sensitivity analysis subset. X-tile software again yielded an optimal cutoff of 11 months (*p* < 0.01) (Figure 2b). Therefore, the optimal temporal threshold for distinguishing EMLM from LMLM was identified as 11 months post-CRC surgery in this study.

### 3.3. Comparative Analysis of EMLM and LMLM

**Figure 3:**
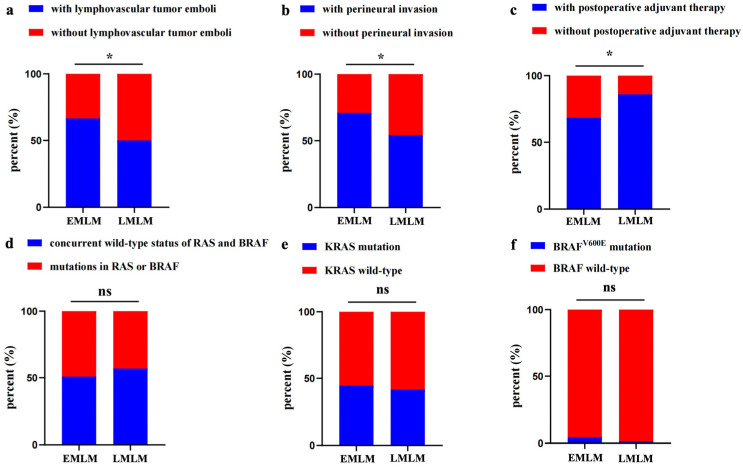
Comparisons between the two groups when patients were grouped according to the 11-month cutoff. (**a**–**c**) Three clinicopathological variables showing statistically significant differences between the two groups. (**d**–**f**) Comparison of RAS/BRAF gene status between the two groups. (*: *p* < 0.05; ns: no significant). **Abbreviations**: EMLM, early metachronous liver metastasis; LMLM, late metachronous liver metastasis.

Using the 11-month cutoff, the 186 patients were divided into the EMLM group (n = 114) and the LMLM group (n = 72). Comparative analysis between the two groups revealed statistically significant differences in lymphovascular tumor emboli (*p* = 0.024), perineural invasion (*p* = 0.019), and postoperative adjuvant therapy (*p* = 0.006) (Figure 3a–c). The difference in the concurrent wild-type status of RAS and BRAF, KRAS mutation, and the BRAF^V600E^ mutation between the two groups was not statistically significant (*p* > 0.05) (Figure 3d–f). A comprehensive comparison of clinicopathological data between the EMLM and LMLM groups is provided in Table 2.

### 3.4. Factors Associated with the Time to Onset of Liver Metastasis

**Figure 4:**
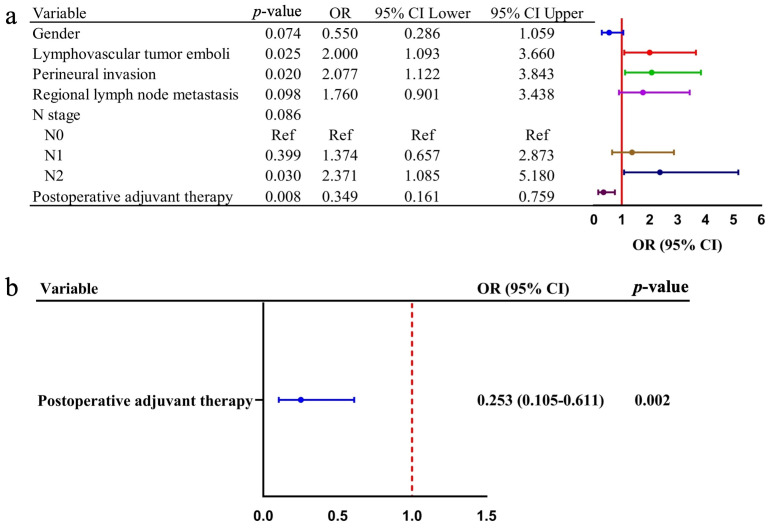
Forest plot of variables with statistical significance. (**a**) The results of the univariable analysis indicated that six variables were statistically significant. (**b**) Further multivariable analysis revealed that one variable remained statistically significant. **Abbreviations**: OR, odds ratio; CI, confidence interval.

Univariable logistic regression analysis was conducted, and the results showed that the variables with statistically significant differences (*p* < 0.1) included gender (OR = 0.550, 95% CI: 0.286–1.059, *p* = 0.074), lymphovascular tumor emboli (OR = 2.000, 95% CI: 1.093–3.660, *p* = 0.025), perineural invasion (OR = 2.077, 95% CI: 1.122–3.843, *p* = 0.020), regional lymph node metastasis (OR = 1.760, 95% CI: 0.901–3.438, *p* = 0.098), N stage (*p* = 0.086), and postoperative adjuvant therapy (OR = 0.349, 95% CI: 0.161–0.759, *p* = 0.008) (Figure 4a). To evaluate the association between KRAS/BRAF^V600E^ mutations and the timing of liver metastasis, we additionally performed a clinically prespecified forced-entry multivariable model in which the two mutations were retained irrespective of their univariable significance. Other variables with *p* < 0.1 in the univariable analysis were also entered into the multivariable logistic regression model. Collinearity assessment using VIF showed no significant collinearity (all VIF < 2). In the final multivariable analysis, neither KRAS mutation (OR, 1.185; 95% CI: 0.641–2.190; *p* = 0.587) nor BRAF^V600E^ mutation (OR, 2.836; 95% CI: 0.302–26.642; *p* = 0.363) was independently associated with the timing of liver metastasis. However, postoperative adjuvant therapy (OR = 0.253, 95% CI: 0.105–0.611, *p* = 0.002) demonstrated a statistical association with the likelihood of LMLM (Figure 4b). The results of univariable and multivariable analyses are detailed in Table 3.

## 4. Discussion

In this study, 11 months post-CRC surgery was identified as the optimal cutoff for differentiating EMLM versus LMLM. No statistically significant association was observed between KRAS/BRAF^V600E^ mutations and the timing of liver metastasis, whereas postoperative adjuvant therapy was statistically associated with the likelihood of LMLM in this cohort. To the best of our knowledge, this is the first study to define a survival-derived optimal temporal cutoff for distinguishing EMLM from LMLM, and to systematically evaluate whether KRAS/BRAF^V600E^ mutations alongside other potential factors are associated with the time to onset of liver metastasis.

The 11-month threshold closely aligns with the 12-month threshold established by the Multisocietal European Consensus on CRLM [22]. It should be noted, however, that the consensus defines the 12-month cutoff based on the date of primary tumor diagnosis rather than the surgical date. Since most patients who develop MLM present with advanced-stage CRC and may undergo varying durations of neoadjuvant therapy—often up to three months—between diagnosis and surgery [23], a one-month discrepancy between these two definitions is entirely expected and can be regarded as equivalent. However, because our cutoff is exploratory and has not undergone external validation, the interpretation and generalization of this cutoff value should be cautious.

In our study, KRAS mutations were detected in 81 patients (43.5%) and BRAF^V600E^ mutations in 6 patients (3.2%), with prevalence rates generally consistent with those reported in previous studies [15,24,25]. Previous studies have revealed that the KRAS mutation is associated with a poor prognosis in CRC patients, particularly in the metastatic setting [13,15,26]. Subsequent research further suggested that the KRAS mutation serves as an independent prognostic factor in CRLM patients [24]. Some studies have even confirmed that the KRAS mutation is an important risk factor affecting the development of liver metastasis after CRC surgery [27,28]. In addition, the BRAF mutation has also been identified in prior work as an independent risk factor for unfavorable outcomes [16,29,30]. A pooled analysis of multiple clinical trials indicated that patients harboring the BRAF^V600E^ mutation exhibit shorter OS and progression-free survival (PFS) compared to those with wild-type BRAF [31]. Some research further revealed that the BRAF^V600E^ mutation emerged as a powerful, independent risk factor for the occurrence of MLM [27,32]. Importantly, our study adds to this understanding by demonstrating that KRAS/BRAF^V600E^ mutations are not significantly associated with the timing of liver metastasis, thereby substantially addressing a gap in this area of research. Nevertheless, the association between the BRAF^V600E^ mutation and the timing of liver metastasis must be interpreted with caution, given the small number of BRAF-mutated cases (n = 6) and the wide confidence interval (OR, 2.836; 95% CI: 0.302–26.642).

Because this study included only patients who had already developed liver metastasis after CRC surgery, our findings pertain to factors associated with the timing of metastasis rather than to risk factors for developing liver metastasis. A clear appreciation of this distinction is essential for the appropriate interpretation of the results. Given that most patients with MLM present with advanced CRC, postoperative adjuvant therapy—including chemotherapy, radiotherapy, immunotherapy, and targeted therapy, used alone or in combination—is routinely recommended in clinical practice [23,33]. With the ongoing evolution of drug regimens, technological advancements, and refinements in multidisciplinary treatment (MDT) concepts, it is reasonable to expect that postoperative adjuvant therapy would play a significant role in delaying or preventing the development of liver metastasis after CRC surgery [23,34]. Paradoxically, however, our study found a statistically significant association between postoperative adjuvant therapy and the likelihood of LMLM, a finding that appears contradictory to this expectation. In fact, this finding precisely reveals the multifaceted roles that adjuvant therapy may exert in the postoperative setting.

First, the primary role of postoperative adjuvant therapy is to destroy the microscopic metastases that remain in the body after surgery. For microscopic foci that are sensitive to postoperative adjuvant therapy, complete eradication can be achieved, thereby reducing the overall incidence of postoperative liver metastasis. For microscopic foci that are partially sensitive, although complete elimination is not possible, their proliferation can be significantly inhibited [35]. Consequently, postoperative adjuvant therapy induces a rightward shift in the temporal distribution curve, such that patients who still develop liver metastasis after postoperative adjuvant treatment are statistically predisposed to late-onset metastasis [36].

Second, owing to inherent tumor heterogeneity, not all CRC cells are equally responsive to postoperative adjuvant therapy [37]. While postoperative adjuvant therapy effectively eliminates the majority of actively proliferating CRC cells—the principal contributors to early metastasis—a small subset of drug-resistant or dormant (G0-phase) tumor stem cells may survive therapeutic assault [38]. These cells resemble “dormant seeds,” residing quiescently in the liver microenvironment [39]. Unaffected by postoperative adjuvant therapy, they may be reactivated years after surgery, triggered by declining immune function or local microenvironmental changes (e.g., inflammation), ultimately giving rise to late liver metastasis [40]. Thus, postoperative adjuvant therapy inadvertently “selects” for cells with inherent latent and drug-tolerant characteristics, and the biological attributes of these cells predispose them to cause metastasis at a later rather than an earlier time point.

Third, interference from non-tumor factors should also be considered. Long-term or intensive postoperative adjuvant therapy may cause sinus damage or liver fibrosis. Although tumors are controlled in the short term, the damaged liver microenvironment may produce inflammatory factors during prolonged repair, thereby providing a fertile ground for residual, dormant tumor cells to be reactivated years later [41]. Furthermore, postoperative adjuvant therapy may reshape the immune system, but over time, immune depletion or immunosenescence may become exacerbated, such that the immune system is no longer capable of suppressing residual lesions in the late stage [42].

However, the definition of postoperative adjuvant therapy in this study was relatively broad, and therefore this finding should also be interpreted with caution. Based on the understanding of the dual dimensions of KRAS/BRAF^V600E^ mutations and postoperative adjuvant therapy, future strategies for the prevention and management of MLM are expected to evolve toward deeper precision and dynamic monitoring. At the basic research level, it is necessary to deeply analyze how driver genes synergistically shape the “metastatic potential” and “treatment sensitivity” of tumors, and to explore the key role of the liver immune microenvironment in drug resistance and tumor dormancy [43,44]. At the clinical translational level, circulating tumor DNA (ctDNA)-based monitoring of microscopic residual disease (MRD) represents a promising breakthrough [45]. Such monitoring could capture the amplification of drug-resistant clones after postoperative adjuvant therapy screening in real time, enabling dynamic risk stratification and guiding individualized adjuvant therapy up-staging or down-staging [46]. The ultimate goal is to realize precise decision-making on “when and how to treat”, thereby shifting the paradigm of MLM management from “treating the disease” to “preventing the disease”.

In addition, regardless of tumor type, multidisciplinary integrated-treatment thinking remains a cornerstone in the management of oncological diseases. Recently, the role of Toll-like receptors (TLRs) in liver regeneration after partial hepatectomy has attracted considerable attention, as TLR signaling regulates inflammatory and reparative signals that influence liver recovery [47]. In this context, TLR-mediated modulation of the hepatic microenvironment may indirectly affect metastatic outgrowth and immune responses, an area that merits dedicated exploration in MLM models [48]. Meanwhile, artificial intelligence (AI) and image processing have shown great potential in the diagnosis, treatment, and prognosis of CRLM, particularly in radiomics and deep learning-assisted decision-making, which similarly holds promise for improving the detection of small metastases, predicting treatment responses, and optimizing individualized surveillance strategies [49,50]. Nevertheless, current studies remain largely retrospective, lack prospective validation, and face challenges in model interpretability [51]. Future efforts should focus on integrating multicenter, multimodal data to facilitate the clinical translation of AI in CRLM management.

Several limitations should be considered in this study. First, because our cutoff was derived from the same cohort using an outcome-driven approach, there is a risk of overfitting. External validation in independent cohorts is needed to confirm the generalizability of this threshold. Second, the sample size of the BRAF^V600E^ mutation subgroup was limited (n = 6), attributable both to the low natural prevalence of this mutation and to our deliberate exclusion of patients with extrahepatic metastasis to ensure the clarity of survival endpoint assessment. Moreover, restricted accessibility to genetic testing further constrained patient enrollment. The resulting lack of statistical power reduces the robustness of subgroup estimates and raises the possibility of selection bias. Therefore, while our preliminary findings are informative, they warrant cautious interpretation and require validation in independent, larger patient cohorts. Third, given the wide time span, heterogeneous medical practices across centers, and the complexity and volume of follow-up data, postoperative adjuvant therapy was analyzed as a composite categorical variable in this study. This analytical approach inherently introduces substantial risks of treatment-selection bias, confounding by indication, and immortal-time bias. Furthermore, each treatment modality has distinct clinical indications and mechanisms of action, which may introduce bias and compromise the scientific validity and reliability of our conclusions. Therefore, future investigations should prioritize either single-center cohorts with standardized treatment protocols or prospective trials specifically designed to dissect the independent contribution of each treatment to the timing of metastasis. Finally, owing to time constraints, a more comprehensive investigation was not feasible in the current study; however, further in-depth investigations utilizing single-cell sequencing and spatial transcriptomics would be both valuable and promising in this context.

## 5. Conclusions

In conclusion, this study identifies 11 months post-surgery as an optimal survival-based cutoff for distinguishing EMLM from LMLM, provides a quantifiable metric for refining postoperative risk assessment, and holds promise for personalizing postoperative surveillance protocols—enabling intensified imaging follow-up during the initial high-risk window for patients with early-onset disease. Multivariable analysis demonstrated that KRAS/BRAF^V600E^ mutations were not associated with the timing of MLM, implying that the chronobiology of metastasis may be more heavily influenced by therapeutic interventions and host-related factors than by genomic characteristics of the primary tumor alone. Conversely, postoperative adjuvant therapy was statistically associated with the likelihood of LMLM, underscoring the pivotal role of timely and complete postoperative adjuvant therapy not only in controlling disease progression but also in reshaping the temporal dynamics of hematogenous hepatic dissemination.

Nevertheless, given the retrospective nature of the cohort and the sample size limitations, our findings should be interpreted as exploratory. Prospective, multicenter, and better-designed validation studies with larger sample sizes are needed to confirm the clinical utility of this cutoff and to further elucidate the biological mechanisms that determine the temporal heterogeneity of metastatic occurrence.

## Figures and Tables

**Table 1:** Clinicopathological baseline characteristics of MLM patients.

Variables	MLM (n = 186)
Age (years), mean (SD)	63 (10)
Gender (female:male)	61: 125
Family history, n (%)	10 (5.4)
Concomitant diseases, n (%)	87 (46.8)
CRC location, n (%)	
Right-sided colon	35 (18.82)
Left-sided colon	49 (26.34)
Rectum	102 (54.84)
Preoperative neoadjuvant therapy, n (%)	95 (51.1)
Lymphovascular tumor emboli, n (%)	112 (60.2)
Perineural invasion, n (%)	120 (64.5)
T stage, n (%)	
T1	2 (1.1)
T2	14 (7.5)
T3	88 (47.3)
T4	82 (44.1)
Regional lymph node metastasis, n (%)	139 (74.7)
N stage, n (%)	
N0	47 (25.3)
N1	73 (39.2)
N2	66 (35.5)
General types, n (%)	
Proliferative type	72 (38.7)
Ulcerative type	70 (37.6)
Infiltrative type	44 (23.7)
Histological types, n (%)	
Adenocarcinoma	178 (95.7)
Mucinous adenocarcinoma	8 (4.3)
Differentiation types, n (%)	
Well-differentiated	3 (1.6)
Moderately differentiated	159 (85.5)
Poorly differentiated	24 (12.9)
dMMR/MSI-H, n (%)	5 (2.7)
KRAS mutation, n (%)	81 (43.5)
BRAF^V600E^ mutation, n (%)	6 (3.2)
Concurrent wild-type RAS and BRAF, n (%)	99 (53.2)
Preoperative CEA (ng/mL), median (IQR)	4.57 (2.52–16.41)
Preoperative CA19-9 (U/mL), median (IQR)	15.94 (8.44–27.31)
Postoperative adjuvant therapy, n (%)	140 (75.3)

**Abbreviations**: CRC, colorectal cancer; MLM, metachronous liver metastasis; SD, standard deviation; IQR, interquartile range; dMMR, deficient mismatch repair; MSI-H, microsatellite instability-high; CEA, carcinoembryonic antigen; CA19-9, carbohydrate antigen 19-9.

**Table 2:** Comparison of clinicopathological data between the two groups.

Variables	EMLM (n = 114)	LMLM (n = 72)	*p*-Value
Age (years), mean (SD)	62.49 (9.574)	63.11 (10.544)	0.680
Gender (female:male)	71: 43	54: 18	0.072
Family history, n (%)	8 (7.0)	2 (2.8)	0.360
Concomitant diseases, n (%)	56 (49.1)	31 (43.1)	0.419
CRC location, n (%)			
Right-sided colon	20 (17.5)	15 (20.8)	0.124
Left-sided colon	25 (21.9)	24 (33.3)	
Rectum	69 (60.5)	33 (45.8)	
Preoperative neoadjuvant therapy, n (%)	62 (54.4)	33 (45.8)	0.256
Lymphovascular tumor emboli, n (%)	76 (66.7)	36 (50.0)	**0.024**
Perineural invasion, n (%)	81 (71.1)	39 (54.2)	**0.019**
T stage, n (%)			
T1	2 (1.8)	0 (0.0)	0.200
T2	9 (7.0)	5 (6.9)	
T3	48 (42.1)	40 (55.6)	
T4	55 (48.2)	27 (37.5)	
Regional lymph node metastasis, n (%)	90 (78.9)	49 (68.1)	0.096
N stage, n (%)			
N0	24 (21.1)	23 (31.9)	0.083
N1	43 (37.7)	30 (41.7)	
N2	47 (41.2)	19 (26.4)	
General types, n (%)			
Proliferative type	41 (36.0)	31 (43.1)	0.483
Ulcerative type	43 (37.7)	27 (37.5)	
Infiltrative type	30 (26.3)	14 (19.4)	
Histological types, n (%)			
Adenocarcinoma	109 (95.6)	69 (95.8)	0.943
Mucinous adenocarcinoma	5 (4.4)	3 (4.2)	
Differentiation types, n (%)			
Well-differentiated	0 (0.0)	3 (4.2)	0.140
Moderately differentiated	96 (84.2)	63 (87.5)	
Poorly differentiated	18 (15.8)	6 (8.3)	
dMMR/MSI-H, n (%)	3 (2.6)	2 (2.8)	0.952
KRAS mutation, n (%)	51 (44.7)	30 (41.7)	0.681
BRAF^V600E^ mutation, n (%)	5 (4.4)	1 (1.4)	0.483
Concurrent wild-type RAS and BRAF, n (%)	58 (50.9)	41 (56.9)	0.419
Preoperative CEA (ng/mL), median (IQR)	4.61 (2.38–19.48)	4.42 (2.58–12.25)	0.465
Preoperative CA19-9 (U/mL), median (IQR)	16.11 (9.16–31.89)	15.94 (7.75–23.35)	0.235
Postoperative adjuvant therapy, n (%)	78 (68.4)	62 (86.1)	**0.006**

**Abbreviations**: EMLM, early metachronous liver metastasis; LMLM, late metachronous liver metastasis; SD, standard deviation; CRC, colorectal cancer; IQR, interquartile range; dMMR, deficient mismatch repair; MSI-H, microsatellite instability-high; CEA, carcinoembryonic antigen; CA19-9, carbohydrate antigen 19-9. Bold *p*-values indicate statistical significance (*p* < 0.05).

**Table 3:** Logistic regression analysis of factors associated with the timing of liver metastasis.

Variables	Univariable		Multivariable	
	OR (95% CI)	*p*-Value	OR (95% CI)	*p*-Value
Age (years)	0.994 (0.964–1.024)	0.678		
Gender (female:male)	0.550 (0.286–1.059)	**0.074**		
Family history (yes:no)	2.624 (0.545–12.807)	0.228		
Concomitant diseases (yes:no)	1.277 (0.705–2.312)	0.420		
CRC location		0.127		
Right-sided colon	Ref			
Left-sided colon	0.781 (0.326–1.870)	0.579		
Rectum	1.568 (0.713–3.447)	0.263		
Preoperative neoadjuvant therapy (yes:no)	1.409 (0.779–2.548)	0.257		
Lymphovascular tumor emboli (yes:no)	2.000 (1.093–3.660)	**0.025**		
Perineural invasion (yes:no)	2.077 (1.122–3.843)	**0.020**		
T stage		0.202		
T1, T2 ^1*^	Ref			
T3	1.080 (0.341–3.421)	0.896		
T4	0.589 (0.316–1.098)	0.096		
Regional lymph node metastasis (yes:no)	1.760 (0.901–3.438)	**0.098**		
N stage		**0.086**		
N0	Ref			
N1	1.374 (0.657–2.873)	0.399		
N2	2.371 (1.085–5.180)	0.030		
General types		0.485		
Proliferative type	Ref			
Ulcerative type	1.204 (0.616–2.354)	0.587		
Infiltrative type	1.620 (0.737–3.561)	0.230		
Histological types (mucinous adenocarcinoma:adenocarcinoma)	1.055 (0.244–4.556)	0.943		
Differentiation types (well-differentiated and moderate:poor) ^2*^	0.485 (0.183–1.286)	0.146		
MMR status (dMMR:pMMR)	0.946 (0.154–5.803)	0.952		
KRAS mutation (yes:no)	1.133 (0.624–2.058)	0.681	1.185 (0.641–2.190)	0.587
BRAF^V600E^ mutation (yes:no)	3.257 (0.373–28.460)	0.286	2.836 (0.302–26.642)	0.363
Concurrent wild-type RAS and BRAF (yes:no)	0.783 (0.433–1.418)	0.420		
Preoperative CEA (ng/mL)	1.009 (0.997–1.021)	0.132		
Preoperative CA19-9 (U/mL)	1.002 (0.997–1.007)	0.442		
Postoperative adjuvant therapy (yes:no)	0.349 (0.161–0.759)	**0.008**	0.253 (0.105–0.611)	**0.002**

^1*^ The number of cases with T1 stage in the LMLM group being zero. ^2*^ The number of well-differentiated cases in the EMLM group being zero. Both 1* and 2* experienced complete separation, meaning that a particular category of the independent variable perfectly predicts the outcome of the dependent variable. Under such conditions, an interpretable OR cannot be calculated [20]. Therefore, this study merged the categories causing complete separation with adjacent categories to form a new variable and reanalyzed the data [21]. **Abbreviations**: EMLM, early metachronous liver metastasis; LMLM, late metachronous liver metastasis; OR, odds ratio; CI, confidence interval; CRC, colorectal cancer; MMR, mismatch repair; dMMR, deficient mismatch repair; pMMR, proficient mismatch repair; CEA, carcinoembryonic antigen; CA19-9, carbohydrate antigen 19-9. Bold *p*-values indicate statistical significance (*p* < 0.1 in univariable analysis; *p* < 0.05 in multivariable analysis).

## Data Availability

Access to the datasets utilized and/or analyzed during the current investigation can be obtained by contacting the corresponding authors, subject to reasonable request.

## Supplementary Materials

The following supporting information can be downloaded at: https://www.mdpi.com/article/10.3390/cancers18152445/s1, Table S1: The molecular-testing details.

## Abbreviations

The following abbreviations are used in this manuscript:

AJCC	American Joint Committee on Cancer
AI	Artificial intelligence
CA19-9	Carbohydrate antigen 19-9
CEA	Carcinoembryonic antigen
CI	Confidence interval
CRC	Colorectal cancer
CRLM	Colorectal liver metastasis
ctDNA	Circulating tumor DNA
dMMR	Deficient mismatch repair
EGFR	Epidermal growth factor receptor
EMLM	Early metachronous liver metastasis
IQR	Interquartile range
LMLM	Late metachronous liver metastasis
MDT	Multidisciplinary treatment
MFS	Metastasis-free survival
MLM	Metachronous liver metastasis
MRD	Microscopic residual disease
MSI-H	Microsatellite instability-high
NGS	Next-generation sequencing
OR	Odds ratio
OS	Overall survival
PCR	Polymerase chain reaction
PFS	Progression-free survival
pMMR	Proficient mismatch repair
SD	Standard deviation
SLM	Synchronous liver metastasis
TLR	Toll-like receptor
VEGF	Vascular endothelial growth factor
VIF	Variance inflation factor
