# Supplementary material for: Factors Associated with the Timing of Liver Metastasis After Colorectal Cancer Surgery: A Retrospective Multicenter Study
Source: Cancers (Basel). 2026 Jul 29;18(15):2445. doi: 10.3390/cancers18152445 (PMC13465346; doi:10.3390/cancers18152445)
Supplement: Supplementary file 1 [file cancers-18-02445-s001.zip › cancers-4356217-supplementary.pdf]

Supplementary Table S1. The molecular-testing details

Item	Beijing Center (n=165)	Shenyang Center (n=21)
NGS platform	Illumina NovaSeq 6000	Thermo Fisher Ion Torrent S5 XL
PCR platform	QuantStudio 12K Flex	Cobas z480
NGS panel	KRAS (exons 2–4), NRAS (exons 2–4), HRAS (exons 2–4), BRAF (exon 15), PIK3CA (exons 9 & 20)	Same target genes, but panel version/probe design may differ (not fully standardized)
PCR assays	Only clinically prevalent variants: KRAS and BRAF ^{V600E} mutations	Same as left
NGS	Mutant allele frequency (MAF) ≥2% (average target depth ≥500×)	Same as left
PCR	MAF ≥5% (validated by serial dilution)	Same as left
Patients tested in-house, n (%)	140 (85%)	17 (81%)
Patients sent out for testing, n (%)	25 (15%)	4 (19%)
Unavailable data proportion	~15% (external tests) with all technical parameters missing	~19% (external tests) with all technical parameters missing

Abbreviations: NGS, next-generation sequencing; PCR, polymerase chain reaction; MAF, mutant allele frequency.
